# Clarifying Concepts and Terms in Biodiversity Informatics

**DOI:** 10.4056/sigs.3907833

**Published:** 2013-05-31

**Authors:** John Deck, Katharine Barker, Reed Beaman, Pier Luigi Buttigieg, Gabriele Dröge, Robert Guralnick, Chuck Miller, Éamonn Ó Tuama, Zack Murrell, Cynthia Parr, Bob Robbins, Dmitry Schigel, Brian Stucky, Ramona Walls, John Wieczorek, Norman Morrison, John Wooley

**Affiliations:** 1 University of California at Berkeley, Berkeley, CA USA; 2 Smithsonian Institution, Washington D.C. USA; 3 University of Florida, Gainesville, FL USA; 4 Alfred Wegener Institute Helmholtz Centre for Polar and Marine Research, Bremen, Germany; 5 Freie Universität Berlin, Berlin, Germany; 6 University of Colorado, Boulder, CO USA; 7 Missouri Botanical Garden, Saint Louis, MO USA; 8 Global Biodiversity Information Facility, GBIF Secretariat, Copenhagen, Denmark; 9 Appalachian State University, Boone, NC USA; 10 Smithsonian Institution, Washington, D.C. USA; 11 University of California at San Diego, La Jolla, CA USA; 12 Metapopulation Research Group & Finnish Museum of Natural History, University of Helsinki, Finland; 13 University of Colorado, Boulder, CO USA; 14 iPlant Collaborative, University of Arizona, Tucson, AZ, USA; 15 University of California, Berkeley, CA USA; 16 School of Computer Science, The University of Manchester, Oxford Road, Manchester, UK; 17 University of California at San Diego, La Jolla, CA USA

## Introduction

“If names be not correct, language is not in accordance with the truth of things. If language be not in accordance with the truth of things, affairs cannot be carried on to success.”

- Confucius, Analects, Book XIII, Chapter 3, verses 4-7, translated by James Legge

Two workshops (hereafter described as “workshops”) were held in 2012, which brought together domain experts from genomic and biodiversity informatics, information modeling and biology, to clarify concepts and terms at the intersection of these domains. These workshops grew out of efforts sponsored by the NSF funded Resource Coordination Network (RCN) project for GSC [1] (RCN4GSC, hosted at UCSD, with John Wooley as PI) to reconcile terms from the Darwin Core (DwC) [2] vocabulary and with those in the MIxS family of checklists (Minimum Information about Any Type of Sequence) [3]. The original RCN4GSC meetings were able to align many terms between DwC and MIxS, finding both common and complementary terms. However, deciding exactly what constitutes the concept of a sample, a specimen, and an occurrence [4] to satisfy the needs of *all* use cases proved difficult, especially given the wide variety of sampling strategies employed within and between communities. Further, participants in the initial RCN4GSC workshops needed additional guidance on how to relate these entities to processes that act upon them and the environments in which organisms live. These issues provided the motivation for the workshops described below.

The two workshops drew largely from experiences of the Basic Formal Ontology (BFO) [5] and were led by Barry Smith, State University of New York at Buffalo. We chose to interact with Smith based on his successful interactions with the GSC in developing the Environment Ontology (EnvO) [6] and also, on the ability of BFO to unite previously disconnected ontologies in the medical domain [7]. The first workshop addressed term definitions in biodiversity informatics, working within the BFO framework, while the second workshop developed a prototype Bio-Collections Ontology, dealing with samples and processes acting on samples.

Concurrent with these workshops were two ongoing efforts involving data acquisition, visualization, and analysis that rely on a solid conceptual understanding of samples, specimens, and occurrences. These implementations are included in this report to show practical applications of term clarification. Finally, this report provides a discussion of some of the next steps discussed during the workshops.

## Workshops

### Semantics of Biodiversity Workshop [8], *University of Kansas, Lawrence, Kansas USA, May 16-17, 2012*

The Semantics of Biodiversity (SOB) workshop hosted at the University of Kansas Biodiversity Institute and sponsored by RCN4GSC, Morphbank [9], and BiSciCol [10], brought together a range of domain experts. On the morning of Day 1, Smith gave a background to ontologies, provided analogies from the biomedical domain, and led a discussion of the basic formal ontology (BFO), an upper-level ontology. BFO describes entities that have continuous existence through time (continuants), such as material objects or qualities, as well as entities which have temporal parts and unfold through time (occurrents), such as processes or temporal regions. The afternoon session began with a lesson in building an ontology within the BFO framework. The session then moved to a discussion of ways to distinguish and track individual objects and attributes of objects using instance identifiers and how to merge, or align, ontologies representing differing views on reality.

The morning of the second day featured presentations by John Wieczorek on the Darwin Core Standard, Dag Endresen on a DNA Extension for Darwin Core, Joel Sachs on the TDWG-RDF interest group, and Norman Morrison on a review of EnvO. In the afternoon session of the second day, Smith wrapped up prior discussions with practical guidance: how to re-use ontologies, principles of singular nouns and understandability, and a critique of DwC terms. Of particular interest was a discussion of strategies employed for managing ontologies and term lists, with examples from the Open Biological and Biomedical Ontologies (OBO) [11].

Finally, the third day consisted of break-out groups, which considered the following topics as they related to earlier discussions: test-bed development, scientific names, the development of a BFO/DwC framework, relationship identifiers, and management structures. Each of the groups delivered a final report and action items. Workshop videos (from Days 1 and 2), workshop documents, and agenda are posted online at http://biocodecommons.org/workshops/sob.html.

### *Bio-Collections Ontology Hackathon,* GSC14, Oxford, UK at the Oxford, e-Research Centre, September 19-20, 2012

The Bio-Collections Ontology Hackathon was held in conjunction with GSC14 [12] and located at the Oxford e-Research Centre, Oxford, UK, and was sponsored by RCN4GSC, GSC, Oxford e-Research Centre (OERC) [13], and BiSciCol. The purposes of this workshop were to undertake a formal definition of samples and sampling processes, formalize the concepts outlined at the SOB workshop as an ontology, and introduce Protégé [14] as a useful ontology editing tool.

Ramona Walls began the workshop by giving an introduction to Protégé, so participants could follow the later discussions by directly coding elements themselves. Participants followed along on their laptops while Walls gave practical tips on using Protégé, covering core terms from the SOB workshop.

On the second day of the hackathon, the term “sample” was considered, using BFO, OBI [15], DwC terms, and MIxS checklists to inform possible meanings and use. Using BFO as a conceptual guide, participants drew on available ontologies to construct a draft ontology encompassing samples and sampling processes. Editing was undertaken in Protégé and a draft ontology was completed at the end of the second day and posted at http://code.google.com/p/biocode-commons/. Samples were classified as “material entities” (from BFO); sampling processes were classified under “processes” (from BFO), including the following processes that could act on samples: collecting, identification, observing, physical extraction, selecting, submitting, and creating information artifact representations (audio recordings, photographs). Other processes we considered, requiring further work to classify, included data sampling, statistical sampling and creating material representation of material entities (casts).

Finally, the group considered the relationship of this ontology to OBI, EnvO, and the Population and Community Ontology (PCO) [16] with discussions about either including the Bio-Collections Ontology within OBI or considering it as a standalone implementation. Trish Whetzel spoke briefly about the National Center for Biomedical Ontology (NCBO) [17] and offered the use of NCBO’s BioPortal [18] to store the Bio-Collections Ontology and other biodiversity related information schemas.

## Standards: Extensions and reference implementations

Ultimately, the goal for work on term definitions and relationships is to enable practical applications for biodiversity science. Two initiatives presented here were being developed concurrently, and both benefited from the outcomes of the workshops. The first effort, the Darwin Core DNA and Tissue Extension aims to track DNA extracts, tissues, and environmental samples as they relate to occurrence records, harvested by the Global Biodiversity Information Facility (GBIF) [19]. Darwin Core per se is essentially an independent implementation of a set of terms and their definitions. Thus, this effort is an extension of the DwC vocabulary combined with a reference implementation. The second effort, BiSciCol, is a linked data project supported by NSF with a goal of tracking specimens, their derivatives, and processes acting on these specimens, across distributed databases. The former implementation relies on term clarification to support development while the latter benefited from using an upper-level ontology to guide classification and the relationship of instances on the semantic web.

### Darwin core DNA and tissue extension

The DNA Bank Network [20] is funded by four German natural history institutions and supported by the German Research Foundation (DFG). It is currently the only portal that provides biodiversity tissue and DNA data in a standardized way and offers interoperability with a wide range of GBIF compliant data sources. The DNA Bank Network is one of the founders of the Global Genome Biodiversity Network (GGBN) [21] and will host and coordinate the GGBN’s planned data portal. While the DNA Bank Network is fully functional, the current framework primarily works with BioCASe [22]/ABCDDNA [23] and not with DwC Archives [24] (DwC Archives being an approach most GGBN partners use to deliver data to GBIF). In addition, the ABCDDNA data model has gaps relative to the needs of GGBN partners. Since the DwC vocabulary contains no DNA or tissue specific classes, there is a need for a DwC DNA and Tissue Extension to address this.

Discussions of how to practically add DNA, tissues, and sequence accession numbers to DwC Archives have developed over the past year, beginning with a meeting in Oxford in February, 2012 [25], continuing with a meeting at TDWG2012 in Beijing, and a conference call in December between GGBN, GBIF, and DwC as well as ABCDDNA architects.

Two primary use cases were considered during this series of meetings on the proposed DwC DNA and Tissue Extension: 1) barcoding, producing a 1:1 mapping between sample and taxonomy, and 2) metagenomics / molecular community ecology that employs next-generation sequencing methods where there is typically a 1-to-many mapping between sample and taxonomy. An important distinction made over both workshops was to consider “sample” exclusive of the DwC term “occurrence”. Samples can potentially contain many discrete organisms, while occurrence is generally regarded as an instance of one organism, known generally by a single taxonomic name or operation identifier. Thus, while occurrence is suitable for representing use case #1, it fails in representing use case #2, especially in the context of reference implementations.

In the interests of timing the first release of a DwC DNA and Tissue Extension, and working with GBIF developers on the follow-up conference call in December of 2012, the group decided to solve use case #1 (1:1 mapping between sample and taxonomy) now by using occurrence as an organizing concept, and then solve use case #2 (bulk sampling) later in 2013. This allows the DwC DNA and Tissue Extension to be immediately useful in linking occurrence data to tissues for single taxon instances, which works seamlessly for GBIF’s harvesting tools. The 1:many case for bulk sampling will be implemented when we can officially recognize samples as a different conceptual unit than occurrence. Advocating proposed changes to DwC vocabulary items to reflect this distinction is part of RCN4GSC’s continuing work in 2013.

### BiSciCol: Tracking identifiers and content inbBiological sciences collections

BiSciCol is building an infrastructure for tracking biological science collections objects and their derivatives. Developing this infrastructure in practice has led to two significant challenges: 1) implementing stable, globally unique, resolvable identifiers, and 2) classifying and linking information across multiple domains and information standards. The ontological approach undertaken in the workshops has significantly helped BiSciCol address the second challenge.

BiSciCol is concerned with tracking objects and their derivatives, regardless of the database source or standards alignment. For example, how can we express a relationship between a specimen, a photo of a specimen, and derived sequence (including laboratory workflows) if each of these entities is expressed using different standards and implementations? Further, how do we generically represent the relationships between samples and processes acting on samples?

By using upper-level ontologies for clarifying the basic nature of objects, we can understand how to relate concepts across various standards, simplifying some classification and terminological challenges. Choosing BFO to structure content for this exercise means we can classify specimens, as a type of “material entity” with a particular “role”, along with derived tissues and DNA, which are “material entities”. The relationship between these objects, while defined by different standards in different places, can be expressed using the transitive “derives_from” relationship term in the Relation Ontology (a BFO project). This allows us, for example, to infer that a specimen and DNA extract share the same “collecting process” (or collecting event) that the specimen was derived from, enabling the plotting of all material or derived material on a world-map based on information discovered through the chain of relationships (assuming the original collecting event happened in nature, not in a lab). The nature of other types of relationships between instance identifiers, such as that between agents and identification instances, can be expressed using non-transitive predicates, enabling further inferences to be made.

The net result for BiSciCol is a clear method for determining allowable relationships and traversing graph-based data derived from multiple standards for biological collections. The BiSciCol project has since developed a list of 4 predicates and 20 concepts at http://biscicol.org/terms/index.html. BiSciCol plans to interoperate with the Open Annotation Ontology Data Model Community Specification for representing these relationships on the semantic web [26]. Continuing to clarify terms and definitions, and building reusable ontologies will greatly assist BiSciCol, and other projects relying on linked data technologies, to manage, track, and analyze biodiversity information in ways not currently possible.

## Next steps

Experiences from these workshops and reference implementations illustrate the utility of concept and term clarification. More work is needed, however, to align terminologies and ontologies and to stabilize term semantics. During the course of the workshops, the following concerns were highlighted. These concerns are not intended as an exhaustive list, or necessarily recommendations from the authors, but merely a record of possible focus areas that workshop participants suggested could be developed further.

### DwC clarifications

More work on the DwC vocabulary is needed to refine terms and term definitions, following guidelines and advice from Smith in the SOB workshop for structuring definitions. A more ambitious goal is to use an upper-level ontology approach to create core, recognized DwC classes. Currently, DwC is in a limbo state where no official classes are recognized (e.g., properties have no domains) but there is a loose arrangement of terms into “categories”. Two options for moving forward are to move DwC towards an official ontology or to transition composite DwC terms into a new ontological framework.

### MIxS as RDF

The MIxS standard exists as a family of check-lists. Mapping terms to RDF with specific URIs for each term is necessary for providing this vocabulary to a broader linked data community.

### GBIF indexing update

The GBIF indexer works around a notion of occurrences as distinct things related to a single taxon. Enabling integration with bulk sampling scenarios and the relationship of many taxa to one sample requires a new way of thinking about the core data types and consequently, the indexing routines used to harvest data from DwC Archives.

### Governance

The current governance ecosystem has a tenuous structure maintained by informal networks of active volunteers. The need for governance structures must be embraced by the community and agreements must be forged in order to efficiently harness the developing ecosystem of ontologies for biodiversity informatics. Examination of successful models from other communities (geospatial, biomedical, ecological) offer a starting point for the community to initiate this much needed governance framework.

### Instance identifiers

Resolution management and services for persistent identifiers are needed. It is vitally important that the identifiers are extremely robust, especially in cases where instance identifiers are used to build graphs and connect information across domains. Resolving situations wherein multiple identifiers refer to the same object is an important activity to this end.

### Test beds and use case development

Understanding community-wide use cases and building test beds for working with data and exploring standards as they impact these use cases will help provide context. The TDWG-RDF interest group has begun development on a preliminary list of use cases [27].

### Branding the effort

How does the community brand this effort? There are several domains at play and components of this effort exist partially in other forms. Is this effort branded as a new effort or subsumed by some other entity? [Table 1]

**Table 1:** Workshop Participants

Fullname	Affiliation	Semantics of Biodiversity, Kansas	Bio-Collections Ontology Oxford	DNA Extension	BiSciCol
Katie Barker	Smithsonian Institution			yes	
Vijay Barve	University of Kansas at Lawrence	yes			
Jim Beach	University of Kansas at Lawrence	yes			
Reed Beaman	University of Florida at Gaineseville		yes		yes
Matthiew Bietz	University of California at Irvine		yes		
Stan Blum	California Academy of Sciences	yes		yes	
Shawn Bowers	SONET	yes			
Pier Luigi Buttigieg	Alfred Wegener Institute Helmholtz Centre for Polar and Marine Research, Bremen, Germany	yes	yes		
Nico Cellinese	University of Florida at Gaineseville				yes
John Deck	University of California at Berkeley	yes	yes	yes	yes
Markus Doering	GBIF			yes	
Gabi Droege	Botanical Garden in Berlin		yes	yes	
Dag Endresen	Global Biodiversity Information Facility	yes			
Paul Flemons	Australian Museum			yes	
Alejandra Gandolfo	Plant Ontology, Cornell	yes			
Robert Guralnick	University of Colorado, Boulder				yes
Robert Hanner	BOLD, GBIF Node	yes			
Alyssa Janning	Univ. of Arizona, BiSciCol	yes			
Michelle Koo	Museum of Vertebrate Zoology, UC Berkeley		yes		
Kris Krishtalka	KU, Biodiversity Institute	yes			
John Kunze	California Digital Library	yes			
James Macklin	Agricultural and Agri-Food Canada			yes	
Andrea Matsunaga	UF/iDigBio	yes			
Chuck Miller	Missouri Bot. Garden, TDWG Chair	yes			
Norman Morrison	EnvO, BioVeL, GSC	yes	yes		
Zack Murrell	Appalachian State University			yes	
Gil Nelson	iDigBio, Florida State University	yes			
Éamonn O’Tuama	GBIF		yes	yes	
Cynthia Parr	Smithsonian Institution, EOL	yes			
Sujeevan Ratnasingham	BOLD		yes		
Jai Rideout	Northern Arizona University		yes		
Robert Robbins	UCSD	yes	yes	yes	
Tim Robertson	GBIF			yes	
Phillipe Rocca-Serra	OERC		yes		
Joel Sachs	TDWG RDF Interest Group	yes			
Inigo San Gil	LTER	yes			
Herbert Schentz	Umweltbundesamt GmbH, Austria	yes			
Dmitry Schigel	Finish Museum of Natural History, University of Helsinki			yes	
Mark Schildhauer	NCEAS/SONET	yes			
Lynn Schriml	University of Maryland School of Medicine			yes	
Barry Smith	State Univ. of NY, Buffalo/ OBO	yes	yes		
Peter Sterk	OERC		yes		
Steve Stones-Havas	Biomatters, New Zealand		yes		
Brian Stucky	BiSciCol, CU-Boulder	yes			yes
Andrea Thomer	UIUC - Library Science	yes			
Mellisa Tulig	New York Botanical Garden	yes			
Dave Vieglais	University of Kansas, University of New Mexico	yes			
Ramona Walls	NYBG	yes	yes		
Brian Wee	NEON	yes			
Trish Whetzel	Stanford, Biomed. Inf. Research	yes	yes		
Jame Whitacre	SI	yes		yes	
Greg Whitbread	Australian Nat'l Botanical Garden	yes			
John Wieczorek	VertNet, Darwin Core	yes	yes	yes	
Kevin Richards	Land Care Research, NZ			yes	
Rusty Russell	Smithsonian Institution			yes	
